# BVT.2733, a Selective 11β-Hydroxysteroid Dehydrogenase Type 1 Inhibitor, Attenuates Obesity and Inflammation in Diet-Induced Obese Mice

**DOI:** 10.1371/journal.pone.0040056

**Published:** 2012-07-02

**Authors:** Long Wang, Juan Liu, Aisen Zhang, Peng Cheng, Xiao Zhang, Shan Lv, Lin Wu, Jing Yu, Wenjuan Di, Juanmin Zha, Xiaocen Kong, Hanmei Qi, Yi Zhong, Guoxian Ding

**Affiliations:** 1 Department of Geratology, the First Hospital Affiliated to Nanjing Medical University, Nanjing, Jiangsu Province, People’s Republic of China; 2 Department of Preventive Medicine, Feinberg School of Medicine Northwestern University, Chicago, Illinois, United States America; 3 Department of Pharmaceutical Chemistry, China Pharmaceutical University, Nanjing, People’s Republic of China; Virginia Commonwealth University, United States of America

## Abstract

**Background:**

Inhibition of 11β-hydroxysteroid dehydrogenase type 1 (11β-HSD1) is being pursued as a new therapeutic approach for the treatment of obesity and metabolic syndrome. Therefore, there is an urgent need to determine the effect of 11β-HSD1 inhibitor, which suppresses glucocorticoid action, on adipose tissue inflammation. The purpose of the present study was to examine the effect of BVT.2733, a selective 11β-HSD1 inhibitor, on expression of pro-inflammatory mediators and macrophage infiltration in adipose tissue in C57BL/6J mice.

**Methodology/Principal Findings:**

C57BL/6J mice were fed with a normal chow diet (NC) or high fat diet (HFD). HFD treated mice were then administrated with BVT.2733 (HFD+BVT) or vehicle (HFD) for four weeks. Mice receiving BVT.2733 treatment exhibited decreased body weight and enhanced glucose tolerance and insulin sensitivity compared to control mice. BVT.2733 also down-regulated the expression of inflammation-related genes including monocyte chemoattractant protein 1 (MCP-1), tumor necrosis factor alpha (TNF-α) and the number of infiltrated macrophages within the adipose tissue *in vivo*. Pharmacological inhibition of 11β-HSD1 and RNA interference against 11β-HSD1 reduced the mRNA levels of MCP-1 and interleukin-6 (IL-6) in cultured J774A.1 macrophages and 3T3-L1 preadipocyte *in vitro*.

**Conclusions/Significance:**

These results suggest that BVT.2733 treatment could not only decrease body weight and improve metabolic homeostasis, but also suppress the inflammation of adipose tissue in diet-induced obese mice. 11β-HSD1 may be a very promising therapeutic target for obesity and associated disease.

## Introduction

Central obesity, an established risk factor for human health, plays an important role in the pathogenesis of insulin resistance, type 2 diabetes, hypertension and cardiovascular disease [1], [2]. However, the underlying mechanisms remain largely unknown [3], [4]. Recently, it has been suggested that amplification of glucocorticoid action within the adipose tissue by the intracellular enzyme 11β-hydroxysteroid dehydrogenase type 1 (11β-HSD1) is involved in the development of central obesity [5], [6], [7], [8], [9]. 11β-HSD1 increases intracellular glucocorticoid levels by converting inert 11-dehydrocorticosterone (cortisone in humans) to active corticosterone (cortisol) in adipocyte and macrophages [10], [11]. Studies performed in model animals and humans have shown that 11β-HSD1 expression is specifically increased in adipose tissue as a result of obesity. Furthermore, Masuzaki *et al* demonstrated that white adipose tissue (WAT)-specific transgene of 11β-HSD1 could cause visceral obesity, insulin resistance, diabetes, dyslipidemia, and hypertension in mice [8]. In contrast, mice with a targeted disruption of the 11β-HSD1 gene (11β-HSD-1^−/−^ mice) exhibited enhanced glucose tolerance, attenuated gluconeogenic responses, and improved lipid profile [12], [13], [14]. These findings suggest that increased activity of 11β-HSD1 in adipose tissue contributes to dysfunction of adipose tissue and subsequent metabolic derangement.

Recently, inhibition of 11β-HSD1 has emerged as a new therapeutic target for the treatment of obesity and metabolic syndrome [15]. Major pharmaceutical companies are now engaged in a new wave of drug development for selective 11β-HSD1 inhibition [16]. These 11β-HSD1 pharmacological inhibitors can improve insulin sensitivity and ameliorate metabolic syndrome not only in most mouse models but also in human [15], [17], [18].

On the other hand, the profound effects of glucocorticoid on the immune system preclude its widespread use as a therapeutic agent for inflammatory diseases [19]. The magnified glucocorticoid action caused by 11β-HSD1 might serve as an important link at the interface of inflammation and obesity [20]. Furthermore, obesity is associated with a chronic low-grade inflammation state, an important risk factor in cardiovascular disease, which might be caused by adipocyte hypertrophy together with infiltration of macrophages into adipose tissue [3]. Therefore, it is imperative that the effects of 11β-HSD1 inhibitor on the inflammation of adipose tissue be clarified.

The aim of the present study was to examine the effect of BVT.2733, a selective inhibitor of 11β-HSD1, on diet induced obesity with a focus on the expression of pro-inflammatory mediators and macrophage infiltration in adipose tissue in mice. Our data affirm the notion that 11β-HSD1 may be a very promising therapeutic target for obesity and associated disease.

## Results

### Effect of HFD and BVT.2733 on Adiposity and Metabolic Parameters

**Figure 1:**
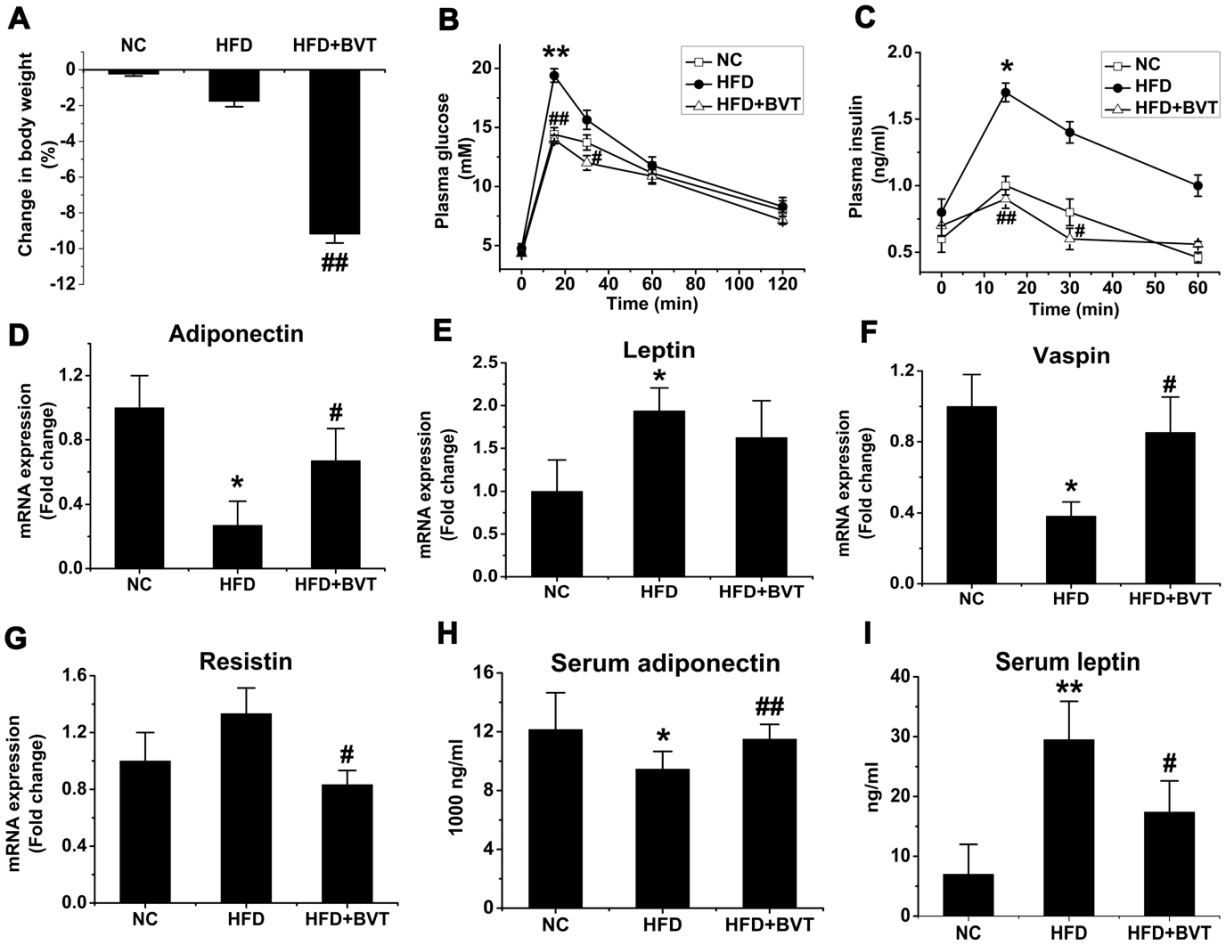
Effect of HDF and BVT.2733 on adiposity and metabolic parameters in C57BL/6J mice. A, Percentage change in body weight. B–C, Glucose tolerance and plasma insulin level. D–G, Changes in adipose gene mRNA expression. H–I, Serum adiponectin and leptin concentration. The results are shown as the means ± SEM. *, *P*<0.05; **, *P*<0.01 compared with NC group; #, *P*<0.05; ##, *P*<0.01 compared with HFD group. n = 5−10 animals per group.

C57BL/6J mice were fed a normal fat diet or HFD for 24 weeks. Mice on HFD showed a significantly higher body weight gain compared with mice on a NC (data not shown). During the last four weeks the HFD-fed mice were treated with BVT.2733 (100 mg/kg, orally) (HFD+BVT mice) or vehicle (HFD mice). The BVT.2733 treatment was not only able to prevent the development of obesity, but also caused rapid weight loss (Fig. 1A). Mice fed on HFD showed glucose intolerance, as evaluated by intraperitoneal glucose tolerance test (Fig. 1B). However, glucose tolerance (Fig. 1B) and insulin levels (Fig. 1C) were improved by BVT.2733 treatment. What’s more, HFD caused marked alterations in the expression of adipokines in adipose tissue including decreased expression of adiponectin (Fig. 1D) and vaspin (Fig. 1F), and increased expression of leptin (Fig. 1E). BVT.2733 administration normalized the expression profile of adiponkines by up-regulating the mRNA levels of adiponectin (Fig. 1D) and vaspin (Fig. 1F) and down-regulating the expression of resistin (Fig. 1G) in adipose tissue. In line with these changes in adipose tissue serum levels of adiponectin (Fig. 1H) and leptin (Fig. 1I) were also improved by BVT.2733 treatment.

### Effect of HFD and BVT.2733 Treatment on the Abundance of Macrophage in Adipose Tissue

**Figure 2:**
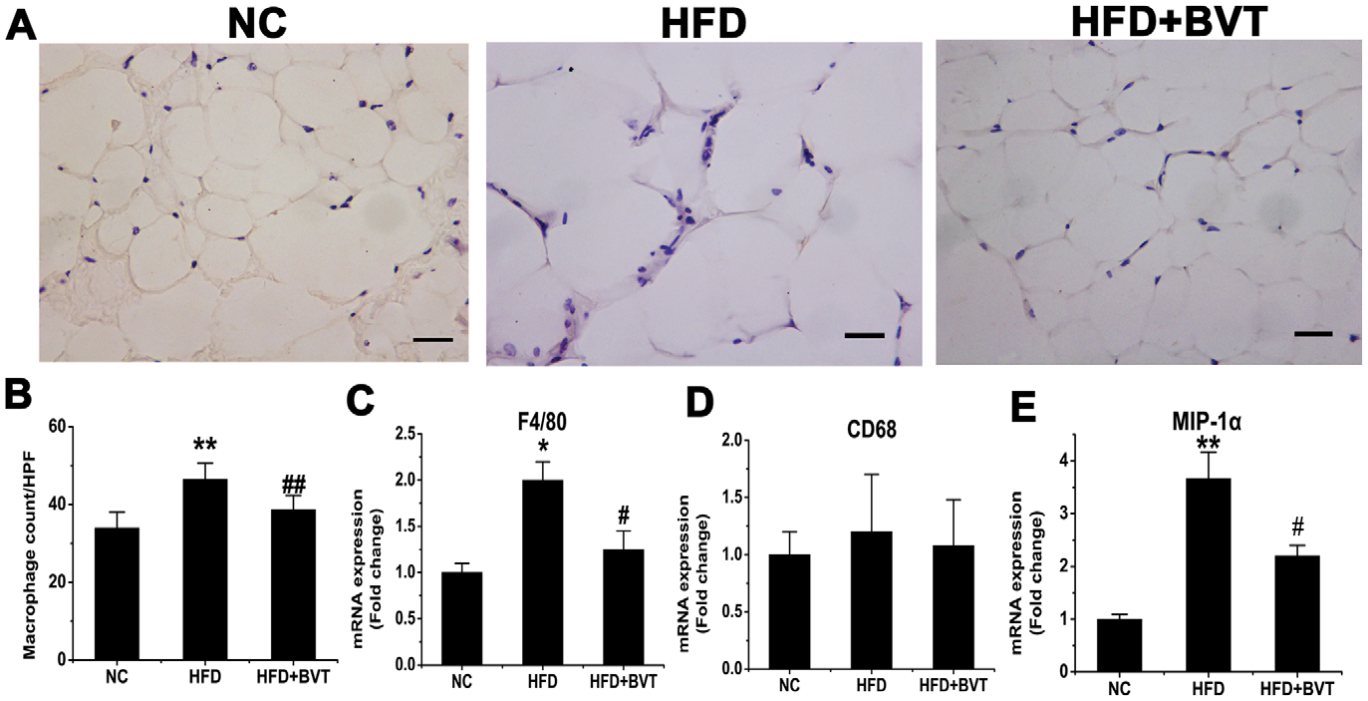
Effect of HFD and BVT.2733 treatment on the abundance of macrophage in adipose tissue. A–B, Representative immunohistochemical staining of white adipose tissue using the specific macrophage marker F4/80 and quantification of the of macrophages present in adipose tissue. C–E, Changes in the expression of macrophage marker genes determined by real time PCR. The results are shown as the means ± SEM. *, *P*< 0.05; **, *P*< 0.01 compared with NC group; #, *P*<0.05; ##, *P*< 0.01 compared with HFD group. n = 6−8 animals per group. Bar  = 50 µm.

**Figure 3:**
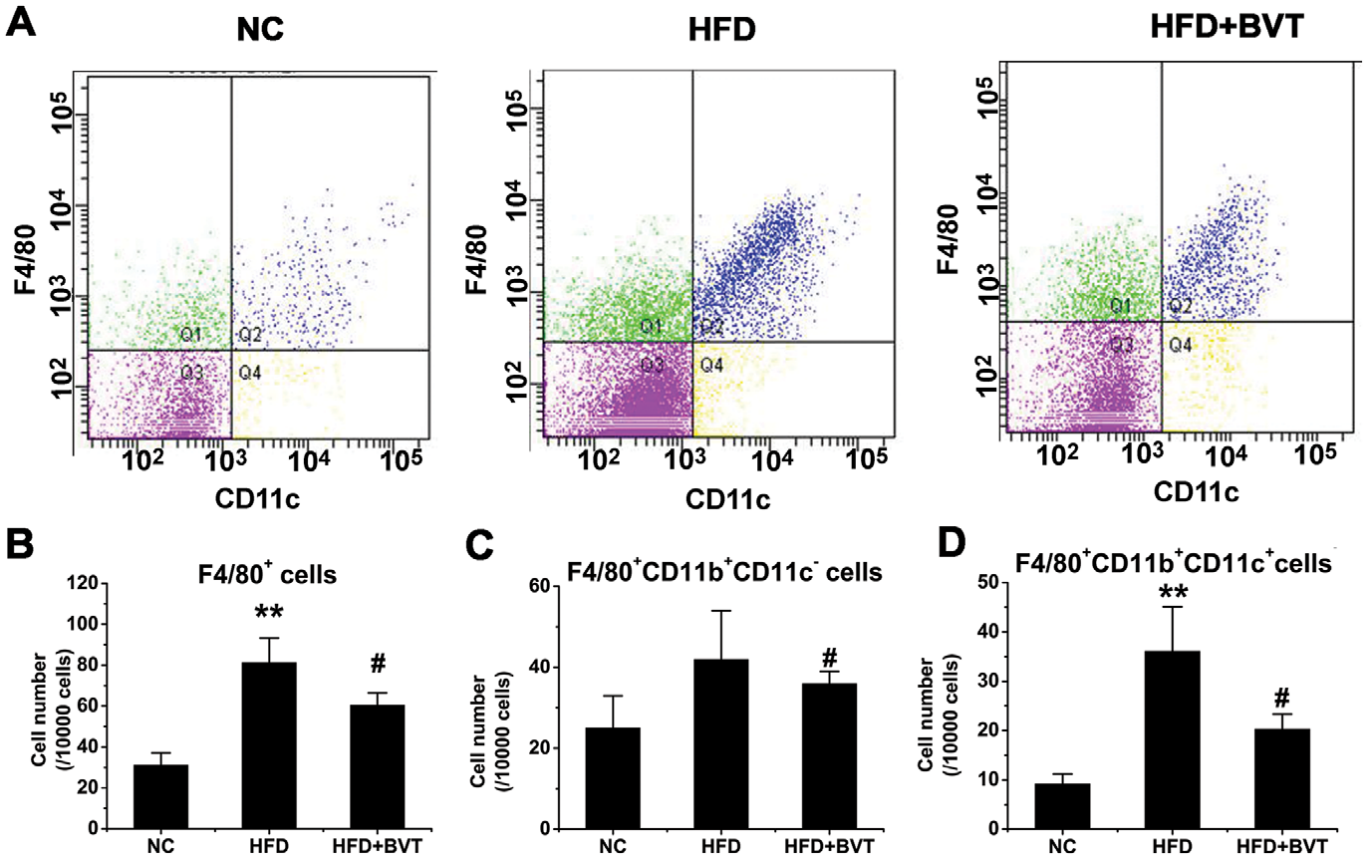
Effect of HFD and BVT.2733 treatment on the number of macrophages in SVF of epididymal fat tissue. Cells in the SVF of epididymal fat tissue from three groups of mice were analyzed using flow cytometry as described in METHODS section. A, Representative flow cytometric profiles of cells in the SVF of epdidymal fat tissue derived from NC, HFD, and HFD+BVT mice individually. B, The cell number in F4/80-positive fraction. C, F4/80-positive/CD11b-positive/CD11c- negetive fraction. D, F4/80-positive/CD11b-positive/CD11c- positive fraction. The results are shown as the means ± SEM. *, *P*<0.05; **, *P*<0.01 compared with NC group; #, *P*<0.05; ##, *P*<0.01 compared with HFD group. n = 3−4 animals per group.

To determine the infiltration of macrophages in adipose tissue after BVT.2733 treatment, immunohistochemistry staining using a specific macrophage marker F4/80 was performed. As shown in Fig. 2A, the number of F4/80 positive macrophages was increased in HFD mice but decreased in HFD+BVT mice. These results were corroborated by quantitative analysis of the number of macrophages present in adipose tissue (Fig. 2B). In agreement with decreased macrophage infiltration, the mRNA expression of macrophage-specific gene F4/80 (Fig. 2C) and macrophage inflammatory protein-1α (MIP-1a) (Figure 2E) was down-regulated in total adipose tissue in HFD+BVT mice compared to HFD mice. However, there was little change in the expression of another macrophage marker gene CD68 between HFD and HFD+BVT mice (Figure 2D). To verify the changes in macrophage infiltration, cells in the stromal vascular fraction (SVF) of epididymal fat tissue from both groups of mice were analyzed by flow cytometry (Figure 3A). The ratio of the F4/80-positive cells was increased in HFD mice, but decreased in HFD+BVT mice (Fig. 3B). F4/80-positive cells were further sorted with anti-CD11c and anti-CD11b antibodies. The data shown that both the CD11b+ CD11c− subset of macrophages (Fig. 3C) and CD11b+ CD11c+ macrophages (Fig. 3D) were decreased in HFD+BVT mice. Together, these data suggest that BVT.2733 treatment decreased macrophage infiltration in adipose tissue in obese mice.

### Effect of HFD and BVT.2733 Treatment on the Inflammation Gene Expression in the Adipose Tissue and the Level of Circulating Inflammation Markers

**Figure 4:**
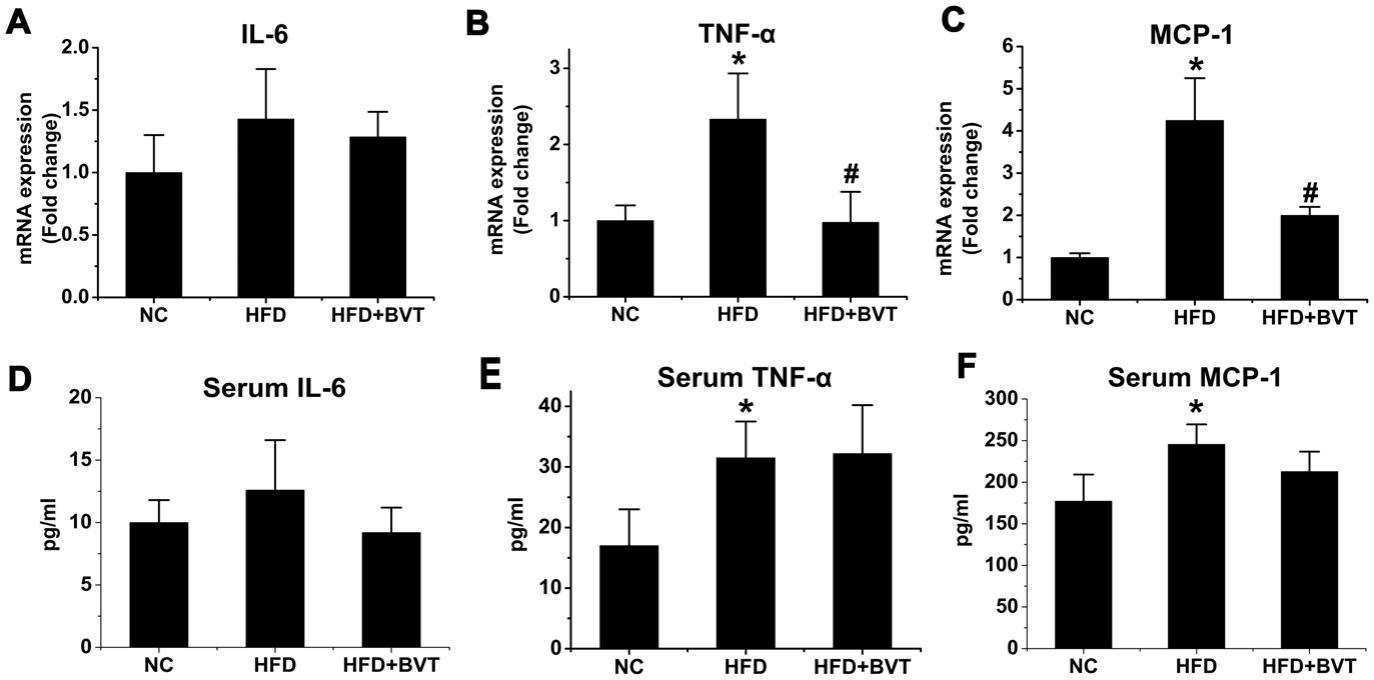
Effect of HFD and BVT.2733 treatment on the inflammation gene expression in the adipose tissue and the level of circulating inflammation markers. A–C, The mRNA expression of IL-6, TNF-α and MCP-1 in adipose tissue. D–E, Plasma concentrations of IL-6, TNF-α and MCP-1. The results are shown as the means ± SEM. *, *P*<0.05; **, *P*< 0.01 compared with NC group; #, *P*<0.05; ##, *P*<0.01 compared with HFD group. n = 5−6 animals per group.

Macrophage infiltration of adipose tissue was increased in HFD mice, and decreased in HFD+BVT mice. Therefore, we investigated the effect of BVT.2733 on the expression and circulating levels of pro-inflammatory cytokines/chemokines. Total RNA was isolated from the whole epididymal fat tissues to determine the expression levels of IL-6, TNF-α and MCP-1 (Fig. 4A, B, C) using real-time RT-PCR. mRNA levels of MCP-1 and TNF-a, but not those of IL-6, were increased in adipose tissue of mice fed a HFD compared to mice fed a NC and BVT.2733 treatment decreased the expression of TNF-α (*P*<0.05, n = 5−6) and MCP-1 (*P*<0.05, n = 5−6). However, plasma concentrations of IL-6, MCP-1 TNF-α (Fig. 4D, E) were not decreased in HFD+BVT mice, suggesting that TNF-α and MCP-1 expressions decreased only locally (within the adipose tissue).

### Effect BVT.2733 on the Inflammation Gene Expression in PA or LPS-activated J774A.1 Macrophages and 3T3-L1 Preadipocyte *in vitro*

**Figure 5:**
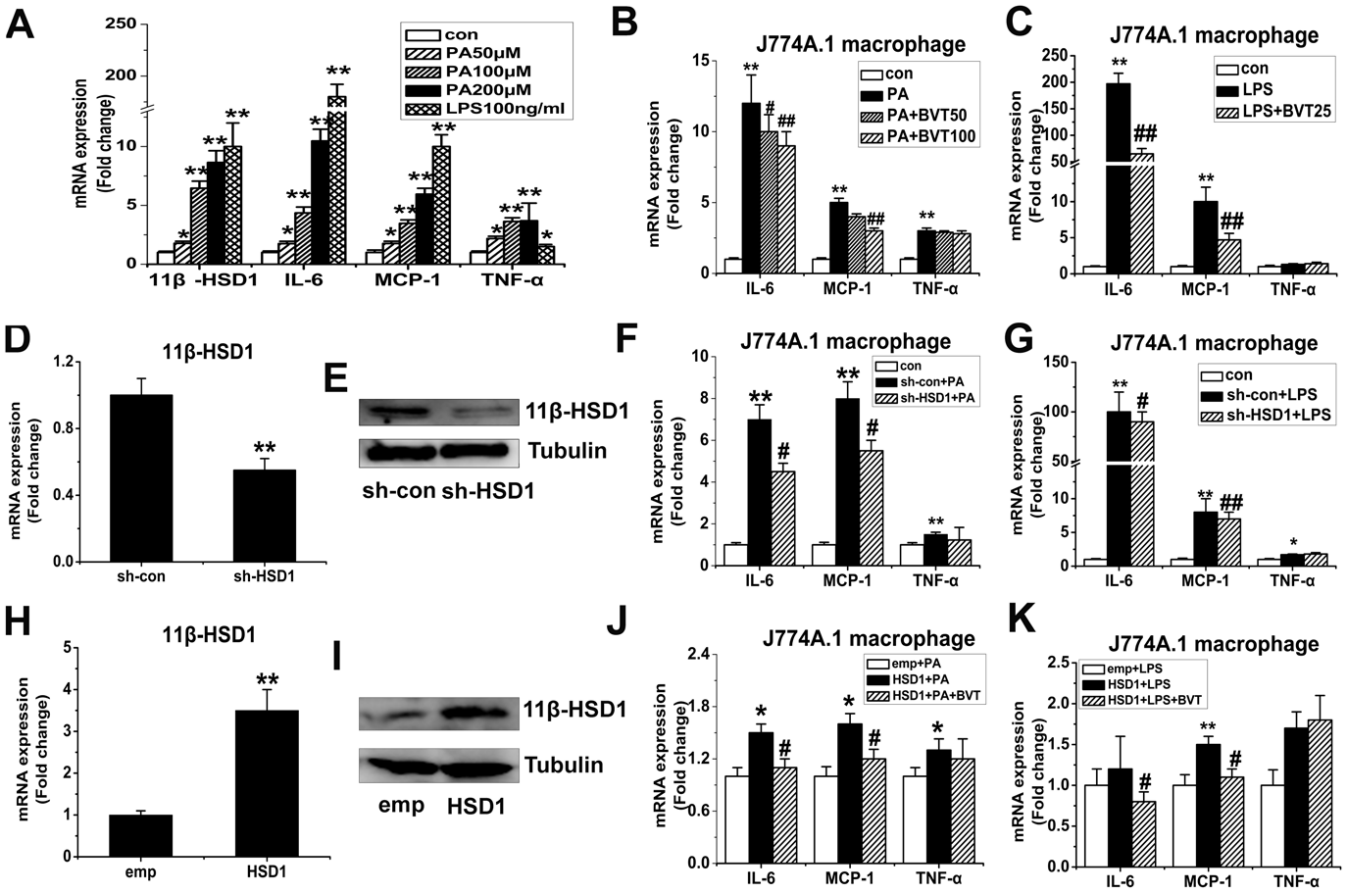
Effect of 11β-HSD1 on the inflammation gene expression in PA or LPS-activated J774A.1 macrophages *in vitro*. A, J774.1 macrophages were treated with palmitic acid (PA) (50−200 µmol/L) or LPS (100 ng/ml) for 24 h. B–C, J774.1 macrophages were activated by PA (200 µmol/L) or LPS (100 ng/ml) and co-treated with 11β-HSD1 inhibitor BVT.2733 (25−100 µmol/L) for 24 h. D–G, J774.1 macrophages were transfected with either sh-RNA for mouse 11β-HSD1 (sh-HSD1) or a negative control (sh-con) by Lentivirus. After 72 h incubation, cells were treated with PA (200 µmol/L) or LPS (100 ng/ml) for 24 h. Efficiency of 11β-HSD1 knockdown on mRNA level (D) and protein level (E). Effects of knockdown of 11β-HSD1 on MCP-1, IL-6, and TNF-α expression in PA (F) or LPS (G) treated macrophages. H–K, J774.1 macrophages were transfected with the expression vector for 11β-HSD1 (HSD1) or a corresponding empty vector (emp) using Lentivirus. After 72 h incubation, cells were treated with PA (100 µmol/L) or LPS (50 ng/ml) and co-treated with 11β-HSD1 inhibitor BVT.2733 for 24 h. Efficiency of 11β-HSD1 overexpression on mRNA level (H) and protein level (I). Effects of overexpression of 11β-HSD1 on MCP-1, IL-6, and TNF-α expression in PA (J) or LPS (K) treated macrophages. mRNA for IL-6, MCP-1 and TNF-α were determined by real-time PCR, protein of 11β-HSD1 were determined by Western blot. The results are shown as the means ± SEM of three individual experiments. *P<0.05; **P<0.01 vs con (B, C, F, G) or sh-con (D) or emp (H) or emp+PA (J) or emp+LPS (K). # *P*<0.05; ## *P*<0.01 vs PA (B) or LPS (C) or sh-con+PA (F) or sh-con+LPS (G) or HSD1+PA (J) or HSD1+LPS (K).

**Figure 6:**
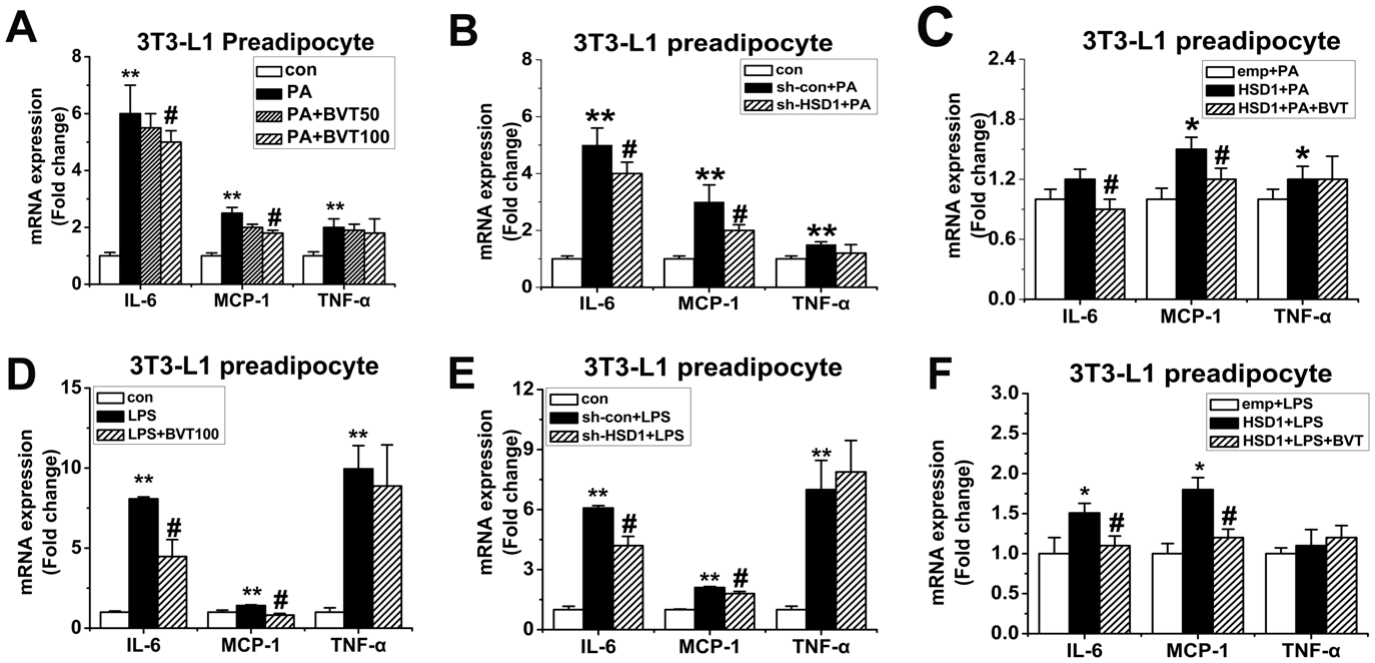
Effect of 11β-HSD1 on the inflammation gene expression in PA or LPS-activated 3T3-L1 preadipocytes *in vitro*. 3T3-L1 preadipocytes were activated by PA (200 µmol/L) (A) or LPS (200 ng/ml) (D) and co-treated with 11β-HSD1 inhibitor BVT.2733 (50−100 µmol/L) for 24 h. 3T3-L1 preadipocytes were transfected with either sh-RNA for mouse 11β-HSD1 (sh-HSD1) or a negative control (sh-con) by Lentivirus. Cells were treated with PA (200 µmol/L) (B) or LPS (200 ng/ml) (E) for 24 h. 3T3-L1 preadipocytes were transfected with the expression vector for 11β-HSD1 (HSD1) or a corresponding empty vector (emp) using Lentivirus. Cells were treated with PA (200 µmol/L) (C) or LPS (200 ng/ml) (F) for 24 h. mRNA for IL-6, MCP-1 and TNF-α were determined by real-time PCR. The results are shown as the means ± SEM of three individual experiments. *P<0.05; **P<0.01 vs con (A, B, D, E) or or emp+PA (C) or emp+LPS (F). # *P*<0.05 vs PA (A) or LPS (D) or sh-con+PA (B) or sh-con+LPS (E) or HSD1+PA (C) or HSD1+LPS (F).

To further assess the effect of BVT.2733 on the expression of inflammation genes in macrophages and preadipocyte, we stimulated murine macrophages (J774A.1) and 3T3-L1 preadipocyte with palmitate (PA) or LPS *in vitro*. mRNA levels of 11β-HSD1 and inflammation genes including IL-6, MCP-1 and TNF-α was increased in macrophages J774A.1 treated with PA or LPS (Fig. 5A). BVT.2733 attenuated the mRNA levels of MCP-1 and IL-6 in PA (Fig. 5B) or LPS (Fig. 5C) treated J774A.1 macrophages. The protein levels of MCP-1 and IL-6 in the medium were also attenuated (Fig. S2A and S2C). However the mRNA levels of TNF-α were not changed after the treatment of BVT.2733. Similar results were observed in 3T3-L1 preadipocyte (Fig. 6A and 6D).

### Effect of 11β-HSD1 Knockdown on Proinflammatory Properties in J774A.1 Macrophages and 3T3-L1 Preadipocytes

To further verify the potential role of 11β-HSD1 in cytokine expression in activated J774A.1 macrophages and preadipocytes, 11β-HSD1 was depleted using lentiviral-based RNAi. When J774A.1 macrophages and 3T3-L1 preadipocytes were infected with 11β-HSD1 shRNA by lentivirus, the expression of 11β-HSD1 was markedly decreased both at mRNA level (Fig. 5D) and the protein level (Fig. 5E). Negative control RNAi did not influence the expression of 11β-HSD1. Consequently, knockdown of 11β-HSD1 in PA (Fig. 5F) or LPS (Fig. 5G)-treated J774A.1 macrophages effectively reduced MCP-1 and IL-6 mRNA levels. Again, the mRNA levels of TNF-α were not changed after the knocked down of 11β-HSD1 in PA (Fig. 5F) or LPS (Fig. 5G)-treated J774A.1 macrophages. Similar results were observed in 3T3-L1 preadipocyte (Fig. 6B and 6E).

### Over-expression of 11β-HSD1 Augmented MCP-1 and IL-6 in PA or LPS-treated J774A.1 Macrophages and 3T3-L1 Preadipocytes

We next examined whether over-expression of 11β-HSD1 by lentiviral infection would be sufficient to drive the augmentation of pro-inflammatory molecules in activated J774A.1 macrophages and preadipocyte. The over-expression efficiencies were confirmed by real-time RT-PCR (Fig. 5H) and Western blot (Fig. 5I) analyses. PA–induced messages of MCP-1, IL-6 and TNF-α were all augmented by 11β-HSD1 (Fig. 5J). The protein levels of MCP-1 and IL-6 in the medium were also augmented (Fig. S2B and S2D). However in LPS-activated macrophage, 11β-HSD1 overexpression only augmented the expression of MCP-1 (Fig. 5K). Furthermore, pharmacological inhibition of 11β-HSD1 by BVT.2733 not only attenuated MCP-1 and IL-6 mRNA levels (Fig. 5J and 5K) but also protein levels (Fig. S2B and S2D) in these cells. Similar results were observed in 3T3-L1 preadipocyte (Fig. 6C and 6F).

### Effect of Glucocorticoid Receptor (GR) on Proinflammatory Properties of 11β-HSD1 in J774A.1 Macrophages

**Figure 7:**
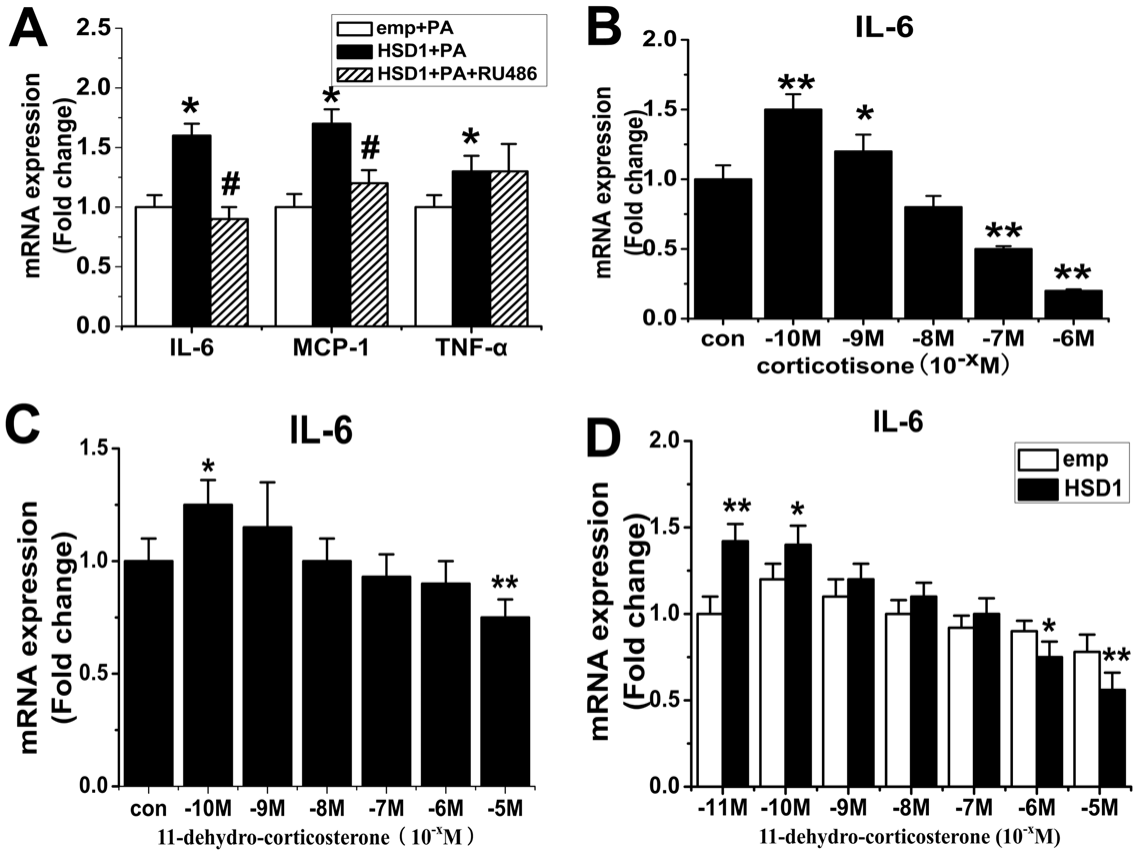
Effect of glucocorticoid receptor (GR) on proinflammatory properties of 11β-HSD1 in J774A.1 macrophages. A, J774A.1 macrophages were transfected with the expression vector for 11β-HSD1 (HSD1) or a corresponding empty vector (emp) using Lentivirus. Cells were treated with PA (100 µmol/L) or co-treated with glucocorticoid antagonist RU486 for 24 h. B, J774A.1 macrophages were activated by LPS(100 ng/ml) in the absence (con) or presence of increasing amounts of corticosterone (10^−10^ M to 10^−6^ M) for 24 h in steroid hormones free media. C, J774A.1 macrophages were activated by LPS (100 ng/ml) in the absence (con) or presence of increasing amounts of 11-dehydro corticosterone (10^−10^ M to 10^−5^ M) for 24 h in Charcoal Dextran Stripped Serum media. D, J774A.1 macrophages were transfected with the expression vector for 11β-HSD1 (HSD1) or a corresponding empty vector (emp) using Lentivirus. Cells were activated by LPS(100 ng/ml) in the absence (con) or presence of increasing amounts of 11-dehydro corticosterone (10^−11^ M to 10^−5^ M) for 24 h in Charcoal Dextran Stripped Serum media. mRNA for IL-6, MCP-1 and TNF-α were determined by real-time PCR. The results are shown as the means ± SEM of three individual experiments. *P<0.05; **P<0.01 vs emp+PA (A) or con (B–C) or emp (D), # *P*<0.05 vs HSD1+PA (A).

To further investigate the mechanism whereby 11β-HSD1 augments inflammation in activated macrophages and preadipocyte, we treated J774A.1 macrophages with glucocorticoid antagonist RU486 in the presence of PA. Surprisingly, we found that RU486 attenuated MCP-1 and IL-6 mRNA levels in activated J774A.1 macrophages (Fig. 7A). Further more, we found that lower concentration corticosterone can increased the inflammation in macrophage (Fig. 7B) in steroid hormones free medium. Normal FBS contains steroid hormones which can act as substrates for HSD1. Therefore, we repeated our experiments with different doses of 11-dehydro-corticosterone in J774A.1 macrophages in Charcoal Dextran Stripped Serum media (steroid hormones free). We found that whereas low concentration 11-dehydro-corticosterone increased the expression of IL-6, higher concentration of 11-dehydro-corticosterone suppressed the expression of IL-6 (Fig. 7C). In addition, J774A.1 macrophages were transfected with the expression vector for 11β-HSD1 (HSD1) or a corresponding empty vector (emp). Cells were activated by LPS (100 ng/ml) in the absence (con) or presence of increasing amounts of 11-dehydro corticosterone (10^−11^ M to 10^−5^ M) for 24 h in Charcoal Dextran Stripped Serum media. When lower concentration 11-dehydro-corticosterone was added in the media, the expression of IL-6 was increased in HSD1 over-expression group, however when higher concentration 11-dehydro-corticosterone was added in the media the expression of IL-6 was suppressed in HSD1 over-expression group (Fig. 7C).

We propose that GCs may exert distinct effects on macrophage function depending on concentration. 11β-HSD1 may augment inflammation by increasing the concentration of local glucocorticoid.

## Discussion

During the last 10 years, evidence has accumulated that strongly argues for an etiological role of 11β-HSD1 in obesity and metabolic syndrome.11β-HSD1 has thus emerged as a major potential drug target for the treatment of obesity and its associated medical conditions. BVT.2733 is a new selective inhibitor of murine 11β-HSD1. In line with previous reports, we report here that inhibition of 11β-HSD1 by BVT.2733 could not only prevent the development of obesity, but also cause rapid weight loss and improve glucose tolerance and insulin levels [15], [17]. What’s more, BVT.2733 treatment for four weeks also caused a marked increase in the expression of adipokines including adiponectin and vaspin in adipose tissue in vivo and we also found that BVT.2733 administration normalized the expression of vaspin (Fig. S1C) and resistin (Fig. S1D) in PA-treated adipocyte *in vitro.* Thus, our findings further confirmed that BVT.2733 could be regarded as an effective agent that ameliorates obesity and metabolic syndrome.

Moreover, growing evidence has asserted that obesity is closely associated with a state of low-grade inflammation in adipose tissue which is characterized by abnormal cytokine production and increased macrophages infiltration [21]. This association has been interpretated as significant in rodents and human studies, and is causally linked to either obesity itself or closely linked diseases such as insulin resistance, type 2 diabetes, and cardiovascular disease [22]. On the other hand, 11β-HSD1 inhibitors might have a harmful effect on adipose tissue by weakening the anti-inflammatory responses of glucocorticoid. In fact, it has been documented that 11β-HSD1^−/−^mice were more susceptible to endotoxemia [23] and 11β-HSD1 played an important role in promoting rapid clearance of apoptotic cells during the resolution of inflammation [24]. Concomitantly, we observed a dramatic decrease in a series of inflammation-related genes including MCP-1 and TNF-α in adipose tissue isolated from HFD+BVT mice compared with HFD mice. We also observed fewer macrophages accompanied by down-regulated expression of a panel of macrophages markers in adipose tissue in HFD+BVT mice. However, macrophages in adipose tissue do not represent a uniform population of cells but rather can exhibit a range of activation states. As reported previously, F4/80, CD11b, and CD11c triple-positive macrophages show very high levels of inflammatory markers. These triple*-*positive cells account for the majority of the increased macrophages content in obese adipose tissue [25]. Therefore, we further analyzed cells in the SVF of epididymal fat tissue from mice using flow cytometry, and we found decreased ratios of F4/80-positive and triple-positive cells with the treatment of BVT.2733. These findings indicate that BVT.2733 may suppress inflammation in adipose tissue via, at least in part, inhibition of cytokine production and lowering the number of macrophages *in vivo.*

It is well known that adipose tissue is a composition of an overwhelming majority of adipocyte, a sizeable majority of preadipocyte, a smaller number of macrophage and a few other cell types [26]. It has been suggested that nonadipocytes (e.g. SV cells) in adipose tissue are the major producers of IL-6 and TNF-α rather than adipocytes [27]. Macrophage infiltration into obese adipose tissue contributes to local and systemic inflammation in subjects with obesity [28]. Recently, it has been suggested that preadipocytes share certain similarities with macrophages in phagocytosis and secretion of inflammatory substances [29]. Our results also indicate that preadipocytes rather than adipocytes play an essential role in LPS-induced cytokine gene expression as shown in (Fig. S1A and S1B). For instance, Ishii-Yonemoto found that TNF-αand IL-1β augmented 11β-HSD1 mRNA expression and activity in 3T3-L1 preadipocytes [30]. Therefore, we employed murine macrophages (J774A.1) and 3T3-L1 preadipocyte for *in vitro* studies. HFD are typically enriched in PA, and excess nutritional intake may increase plasma and tissue PA concentrations [31], [32]. J774A.1 and 3T3-L1 preadipocyte were stimulated with PA or LPS. Interestingly, we found that expression of the inflammation–related genes and 11β-HSD1 were both substantially increased in PA or LPS activated J774.1 macrophages. Consistent with the results *in vivo*, we observed BVT.2733 or 11β-HSD1 knockdown decreased expression levels of various inflammation-related genes, including IL-6 and MCP-1, in activated macrophages. In contrast, over-expression of 11β-HSD1 augmented expression of MCP-1 and IL-6 in activated J774A.1 macrophages and 3T3-L1 preadipocytes. Therefore, our cell-based and animal results revealed that 11β-HSD1 is an important regulator at the interface of obesity and inflammation, and the inhibition of 11β-HSD1 by BVT.2733 could prevent the development of obesity and reduce the inflammation of adipose tissue both *in vivo* and *in vitro*.

Glucocorticoids have been widely used to treat inflammatory diseases for almost 60 years due to the potent anti-inflammatory properties [19], but it has been known to have both permissive and suppressive effects on gluconeogensis, glycogenolysis and lipolysis [33]. However, our studies suggest that slight increase of glucocorticoid by 11β-HSD1 may have a permissive effect on inflammation. Our data support that over-expression of 11β-HSD1 augmented MCP-1 and IL-6 in PA or LPS-treated J774A.1 macrophages and 3T3-L1 preadipocytes. Furthermore, we found glucocorticoid at low concentration can increase the inflammation in macrophage (Fig.7B). Meanwhile, pharmacological inhibition of 11β-HSD1 and RNA interference against 11β-HSD1 reduced the mRNA levels of MCP-1 and IL-6. In addition, the expression of inflammation-related gene augmented by 11β-HSD1 overexpression was also attenuated by BVT.2733 in macrophages and preadipocytes. In fact, Lim et al [34] has also reported that 10^−10^ M of corticosterone enhanced expression of pro-inflammatory cytokines while higher concentrations of corticosterone (10^−7^ M–10^−6^ M) strongly repressed macrophage function. Our data also supported that low concentration 11-dehydro-corticosterone added in the steroid hormone-free media mimics the medium supplemented with 10% heat-inactivated FBS, but when higher concentration of 11-dehydro-corticosterone was added into the media it acts as an anti-inflammatory hormone. Recently, Wamil et al [35] demonstrated that HSD1 deficiency also suppressed inflammation of adipose tissue in obesity. Therefore, it seems that glucocorticoids may exert both positive and negative influence on inflammation dependent on their concentrations. It may open a fresh avenue for molecular and cellular mechanism of adipose inflammation and dysfunction.

In summary, we demonstrate that treatment of a selective inhibitor of 11β-HSD1, BVT.2733, could prevent the development of obesity and reduce inflammation in cells and adipose tissues in DIO mice. Our study suggests that 11β-HSD1 is an important regulator at the interface of obesity and inflammation, and maybe be a promising target of obesity-associated diseases.

## Materials and Methods

### Ethics Statement

All animal and tissue sample experiments were performed in accordance with the guidelines of the National Institutes of Health and Nanjing Medical University with procedures(ID:2082101) approved by the Institutional Animal Care and Use Committee of the university.

### Reagents

BVT.2733, an 11β-HSD1 selective inhibitor was synthesized according to the patent information by China Pharmaceutical University. Anti-F4/80 antibody was purchased form Serotec (Oxford, UK). Phycoerythrin-Cy5 conjugated anti-mouse F4/80 antibody was purchased form eBioscience (San Diego,CA, USA). Phycoerythrin anti-mouse CD11b and Alexa Fluor488 anti-mouse CD11c antibodies were purchased from BioLegend (San Diego,CA). RU486, corticotisone, LPS and PA were purchased from (Sigma-Aldrich, St. Louis, MO). M-MLV, dNTP, RNase inhibitor and other reverse transcription reagents were purchased from Promega Corp (Madison, WI, USA). Trizol were purchased from Invitrogen (Carlsbad, CA, USA).

### Animals

Male C57BL/6J mice at age of 18 days were purchased from Slac Laboratories (Shanghai, China). In all experiments, mice were housed three or four per cage in a room kept at 23±1°C with a 12-h light, 12-h dark cycle and were allowed free access to water and food. From 3 weeks of age, all mice were fed with a normal chow diet containing 10% calory from fat (NC mice) (Collaborative Bio-Engineering Corporation, Nanjing, China), or a high fat diet (HFD) containing 50% calorie from fat (Collaborative Bio-Engineering Corporation, Nanjing, China) for 24 weeks. During the last four weeks the HFD-fed mice were dosed (09.00 and 17.00 h) with BVT.2733 (100 mg/kg, orally) (HFD+BVT mice) or vehicle (HFD mice). Body weight and food intake were determined weekly. At the time of sacrifice, trunk blood was collected, centrifuged, and stored at −20°C. Adipose tissues were weighed, frozen in liquid nitrogen, and stored at −80°C.

### Intraperitoneal Glucose Tolerance Test

The mice were fasted overnight and then injected intraperitoneally with 2 mg/g D-glucose (25% stock solutionin saline). Blood samples were taken by tail venesection at 0 min (before injection and within 1 min of disturbing the cage) and at 15-, 30-, 60-, and 120-min intervals after the glucose load. Glucose was measured with the Accu-Chek Aviva system (Roche, Germany) at 0-, 15-, 30-, 60-, and 120-min post glucose load. Insulin was measured with the Ultra Sensitive Mouse Insulin ELISA kits (Millipore Corporation, MA, USA) at 0-, 15-, 30-, 60-min post glucose load.

### Immunohistochemistry of Macrophages in White Adipose Tissues

Portions of epididymal adipose tissue were removed and fixed with 10% formaldehyde and embedded in paraffin. Sections were stained with hematoxylin and eosin. For the detection of macrophage infiltration in adipose tissue, immunohistochemistry was performed with F4/80 antibody, which detected macrophage-specific protein. The number of F4/80-positive cells was counted in a blinded fashion under a microscope with a 400× objective. More than 50 serial fields were examined in each mouse, and five mice were analyzed per group.

### Isolation of Adipocytes and Stromal-vascular Fractions

Epididymal adipose tissues from mice were rinsed in saline, minced into fine pieces, and digested with collagenase (Sigma-Aldrich, St. Louis, MO) with Krebs-Hense-leit-HEPES buffer, pH 7.4, supplemented with 20 mg/ml of BSA and 2 mmol/l glucose at 37°C using a shaker for 45 min. Then, the samples were passed through a mesh and fractionated by brief centrifugation (1,000 rpm). The pellets or the floating cells were collected as the stromal-vascular fraction (SVF) or as the adipocyte fraction, respectively. Cells in each fraction were used for flow cytometry analysis.

### Flow Cytometry Analysis

Cells in the SVF were incubated with Fc Block (BD Biosciences, San Jose, CA) for 20 min at 4°C prior to staining with fluorescently labeled primary antibodies or control IgGs for 25 min at 4°C. Then cells were gently washed twice and resuspended in FACS buffer with propidiumiodide (Sigma-Aldrich). SVF were analyzed using FACS Calibur and FACS Aria flow cytometers (BD Biosciences). Unstained, single stains, and fluorescence minus one controls were used for compensation and gates settings. The data analysis was performed using FlowJo (Tree Star, Ashland, OR). Macrophages were identified as F4/80-positive, F4/80-positive/CD11b-positive or F4/80-positive/CD11b-positive/CD11c-positive cells, respectively. The numbers of macrophages were shown according to the percentage of each fraction per 10000 cells.

### Serum Analysis

Serum levels of adiponectin and leptin were determined using commercially available ELSA kits from Millipore (Millipore Corporation, MA) and Linco (LINCO Research Inc., St. Charles, MO) individually. Proinflammatory cytokines interleukin-6 (IL-6), monocyte chemoattractant protein-1 (MCP-1) and tumour necrosis factor α (TNF α) were determined using the mouse commercially available kit from Bender (Bender MedSystems, Austria). All assays were performed according to the manufacturer’s protocol.

### Cell Culture

The mouse reticulum cell sarcoma-derived cell, J774.1, was obtained from the American Type Culture Collection (ATCC, Manassas, VA), and maintained in RPMI 1640 supplemented with 10% heat-inactivated FBS(Life Technologies Corporation, USA)in a humidified 37°C/5% CO_2_ incubator. Some J774.1 was maintained in RPMI 1640 supplemented with 10% Charcoal Dextran Stripped Serum (Life Technologies Corporation, USA) as indicated. The mouse fibroblast 3T3-L1 preadipocytes was obtained from ATCC and maintained in DMEM culture medium supplemented with 10% fetal calf serum and 4 mM glutamine.

### Plasmids and Lentiviral Vectors

Full-length mouse cDNA sequence for 11β-HSD1 was cloned into pLL3.7-BSD lentiviral vector. The RNAi construct for 11β-HSD1 was generated using one sequence in the coding region of 11β-HSD1: 5′ -GTACGGAACTGCATAAGCA-3′ (Sigma). Oligonucleotides containing this sequence or random sequence were subcloned into the lentiviral vector.

### Lentivirus Production and Infection of J774A.1 Macrophage and 3T3-L1 Preadipocyte

Recombinant lentiviruses were produced by cotransfecting 293T cells with the lentivirus expression plasmid and packaging plasmids using Lipofectamine 2000 (Invitrogen) according to themanufacturer’s instruction. Infectious lentiviruses were harvested at 48 after transfection and filtered through 0.45 m cellulose acetate filters. The infection of 3T3-L1 preadipocytes and J774A.1 macrophages was carried out by adding lentivirus into the cell culture; the controls were infected with scrambled shRNA or vector. 12 hours after the incubation, the medium was changed. Infected cells were selected with BSD (3 mg/ml).

### Preparation of PA

To avoid bacterial LPS contamination, [36], [37] PA were reconstituted to 10 mmol/L in ethanol, and diluted directly into warmed culture medium for experiments.

### RNA Preparation and Quantitative Real-time PCR

Total RNA was extracted from epididymal adipose tissues using TRIZOL reagent (Invitrogen) according to the manufacturer’s instructions. Two micrograms of total RNA were reverse-transcribed with 200 U Moloney murine leukemia virus reverse transcriptase (M-MLV, Promega, Madison, WI, USA), and in the presence of 0.5 mmol/L deoxynucleotide triphosphate, 25 U RNase inhibitor, and 0.5 µg N15 random primers, in a total volume of 25 µl. PCR primers were designed by Primer5 software. The sequences of the primers used are available upon request. Each quantitative real-time PCR was carried out in triplicate, in a 25 µl volume of SYBR Green Real-time PCR Master Mix (Toyobo, Osaka, Japan). The PCR program was designed as follows: 60 s at 95°C, followed by 40 cycles of 15 s at 95°C, 15 s at 60°C, 45 s at 72°C, and 5 s at 80°C on the plate reader (Rotor Gene-3000, Corbett Research, Sydney, Australia). All data were analyzed using the expression of the gene encoding β-actin as an internal reference. Expression for each gene is arbitrarily set at 1 to facilitate comparison between several groups.

### Western Blot Analysis

For western blot analysis, cells or tissues were lysed in RIPA buffer (0.5% NP-40, 0.1% SDS, 150 mM NaCl, 50 mM Tris-Cl [pH 7.5]). Proteins were separated by SDS-PAGE, transferred to PVDF membrane (Millipore) and probed with anti-11β-HSD1 (Abcam), and anti-Tubulin (Abcam) antibodies.

### Statistics

Comparisons were performed using the Student’s t test for two groups, or ANOVA for multiple groups. Results are presented as means ± SEM. A *p* value of less than 0.05 was considered statistically significant.

## Supporting Information

Figure S1 **Effect of BVT.2733 on the adipokines gene expression in 3T3-L1 adipocyte.** 3T3-L1 preadipocytes were induced to differentiation adipocyte, d0, d4, d8 cells were harvested for the mRNA analyses of MCP-1 (A). Preadipocytes and fully differentiation adipocytes were activated by LPS(0.01–10 µg/ml) separately for 24 h (B). Fully differentiation adipocytes were activated by PA(100 µmol/L) or coatreated with BVT.2733 for 24 h (C), mRNA for MCP-1, Vaspin and Resistin were determined by real-time PCR. The results are shown as the means ± SEM. *, *P*<0.05; **, *P*< 0.01 compared with d0 or con group; #, *P*<0.05; ##, *P*<0.01 compared with PA group.
(TIF)

Figure S2 **Effect of 11β-HSD1 on the inflammation protein levels in medium** ***in vitro*** **.** J774.1 macrophages were activated by PA (200 µmol/L) and co-treated with 11β-HSD1 inhibitor BVT.2733 (50–100 µmol/L) for 24 h, concentrations of MCP-1 (A)and IL-6(C)in the media were measured by ELISA. J774.1 macrophages were transfected with the expression vector for 11β-HSD1 (HSD1) or a corresponding empty vector (emp) using Lentivirus. After 72 h incubation, cells were treated with PA (100 µmol/L) and co-treated with 11β-HSD1 inhibitor BVT.2733 for 24 h; concentrations of MCP-1 (B) and IL-6 (D) in the media were measured by ELISA. The results are shown as the means ± SEM. *, *P*<0.05; **, *P*< 0.01 compared with con or emp+PA group; #, *P*<0.05; ##, *P*<0.01 compared with PA or HSD1+PA group.
(TIF)
