# Supplementary figures and images for: BVT.2733, a Selective 11β-Hydroxysteroid Dehydrogenase Type 1 Inhibitor, Attenuates Obesity and Inflammation in Diet-Induced Obese Mice
Source: PLoS One. 2012 Jul 2;7(7):e40056. doi: 10.1371/journal.pone.0040056 (PMC3388048; doi:10.1371/journal.pone.0040056)

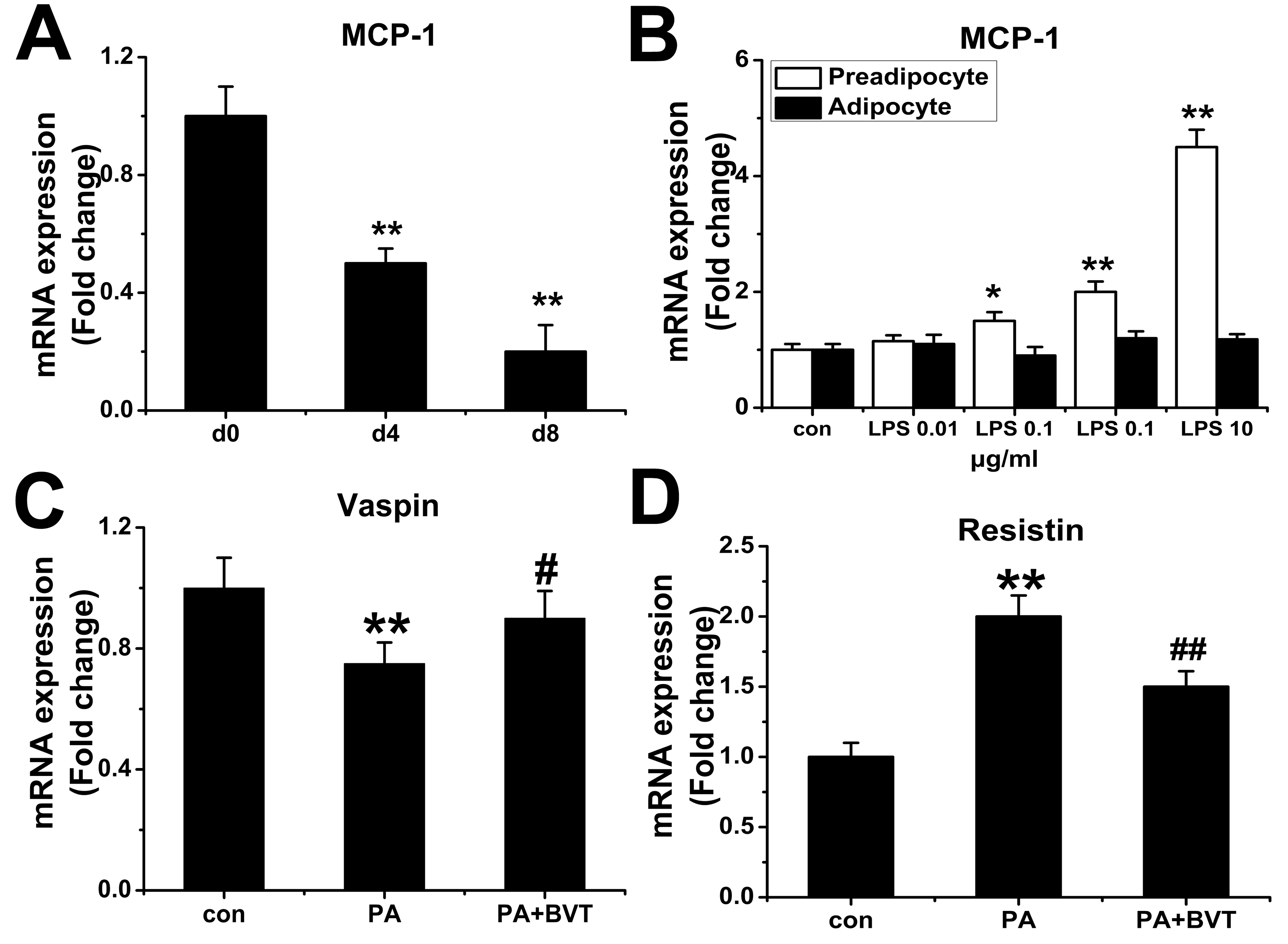

Supplement: Figure S1 — Effect of BVT.2733 on the adipokines gene expression in 3T3-L1 adipocyte. 3T3-L1 preadipocytes were induced to differentiation adipocyte, d0, d4, d8 cells were harvested for the mRNA analyses of MCP-1 (A). Preadipocytes and fully differentiation adipocytes were activated by LPS(0.01–10 µg/ml) separately for 24 h (B). Fully differentiation adipocytes were activated by PA(100 µmol/L) or coatreated with BVT.2733 for 24 h (C), mRNA for MCP-1, Vaspin and Resistin were determined by real-time PCR. The results are shown as the means ± SEM. *, P<0.05; **, P< 0.01 compared with d0 or con group; #, P<0.05; ##, P<0.01 compared with PA group. (TIF) [file pone.0040056.s001.tif]

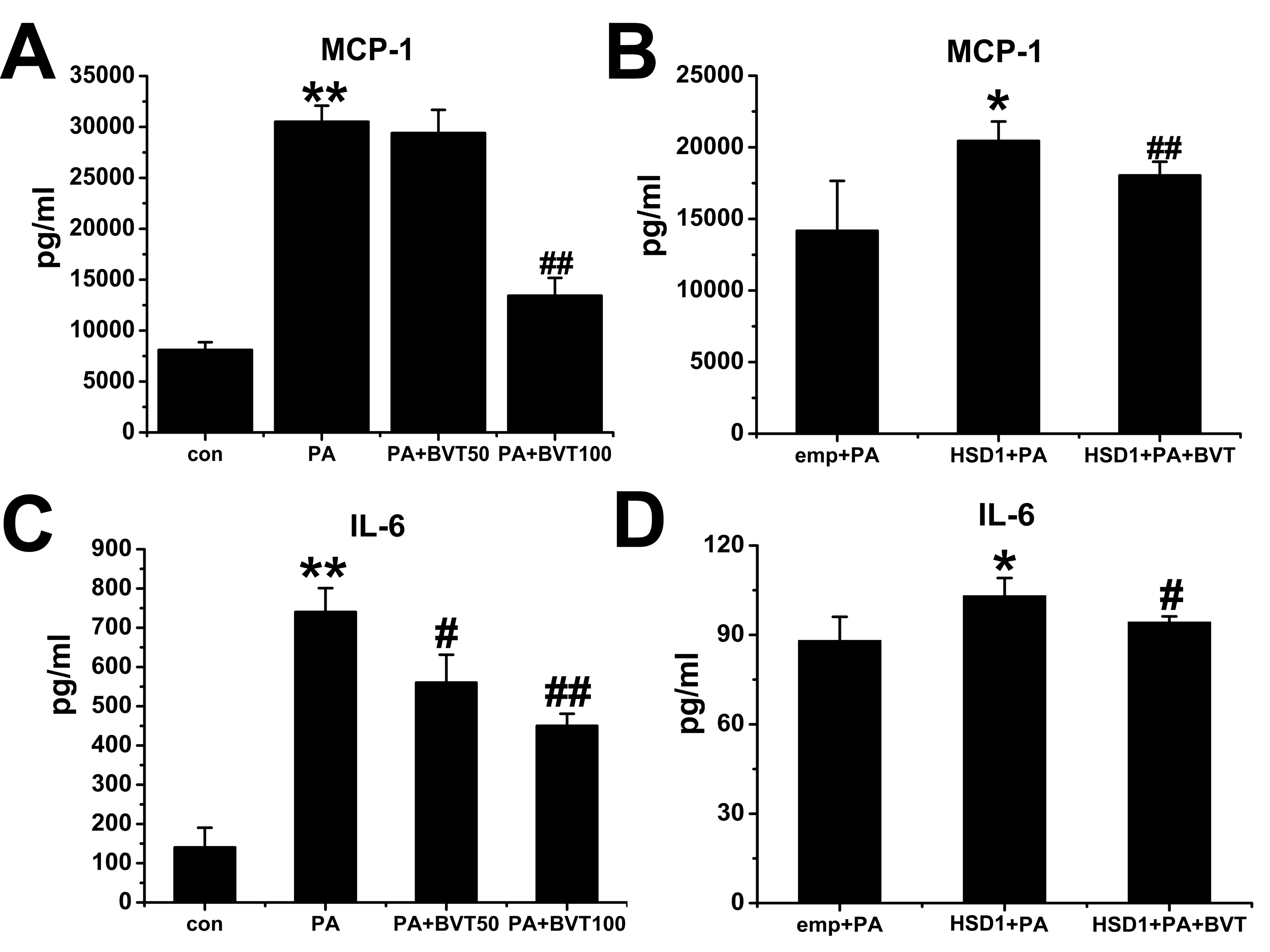

Supplement: Figure S2 — Effect of 11β-HSD1 on the inflammation protein levels in medium in vitro . J774.1 macrophages were activated by PA (200 µmol/L) and co-treated with 11β-HSD1 inhibitor BVT.2733 (50–100 µmol/L) for 24 h, concentrations of MCP-1 (A)and IL-6(C)in the media were measured by ELISA. J774.1 macrophages were transfected with the expression vector for 11β-HSD1 (HSD1) or a corresponding empty vector (emp) using Lentivirus. After 72 h incubation, cells were treated with PA (100 µmol/L) and co-treated with 11β-HSD1 inhibitor BVT.2733 for 24 h; concentrations of MCP-1 (B) and IL-6 (D) in the media were measured by ELISA. The results are shown as the means ± SEM. *, P<0.05; **, P< 0.01 compared with con or emp+PA group; #, P<0.05; ##, P<0.01 compared with PA or HSD1+PA group. (TIF) [file pone.0040056.s002.tif]
